# Nanoparticle-Mediated Hyperthermia and Cytotoxicity Mechanisms in Cancer

**DOI:** 10.3390/ijms25010296

**Published:** 2023-12-25

**Authors:** Vanessa-Meletia Bala, Dimitra Ioanna Lampropoulou, Stamatiki Grammatikaki, Vassilios Kouloulias, Nefeli Lagopati, Gerasimos Aravantinos, Maria Gazouli

**Affiliations:** 1 Hygeia Hospital, Erithrou Stavrou 4, 15123 Marousi, Greece; vanessabalamd@gmail.com; 2 ECONCARE, Chatzigianni Mexi 5, 11528 Athens, Greece; d_lambropoulou@yahoo.gr; 3 Laboratory of Biology, Medical School, National and Kapodistrian University of Athens, 11527 Athens, Greece; matinagramm@hotmail.com (S.G.); nlagopati@med.uoa.gr (N.L.); 4 Radiation Oncology Unit, 2nd Department of Radiology, Attikon University Hospital, Medical School, National and Kapodistrian University of Athens, 11527 Athens, Greece; vkouloul@ece.ntua.gr; 5 Euroclinic, Athanasiadou 7-9, 11521 Athens, Greece; garavantinos@yahoo.gr

**Keywords:** cancer, hyperthermia, nanoparticles, cytotoxicity mechanisms

## Abstract

Hyperthermia has the potential to damage cancerous tissue by increasing the body temperature. However, targeting cancer cells whilst protecting the surrounding tissues is often challenging, especially when implemented in clinical practice. In this direction, there are data showing that the combination of nanotechnology and hyperthermia offers more successful penetration of nanoparticles in the tumor environment, thus allowing targeted hyperthermia in the region of interest. At the same time, unlike radiotherapy, the use of non-ionizing radiation makes hyperthermia an attractive therapeutic option. This review summarizes the existing literature regarding the use of hyperthermia and nanoparticles in cancer, with a focus on nanoparticle-induced cytotoxicity mechanisms.

## 1. Introduction

Cancer is one of the most lethal human illnesses, exhibiting a variety of distinctive clinical symptoms and causing millions of deaths worldwide each year. According to the World Health Organization, it is one of the highest mortality causes globally, accounting for over 10 million fatalities in 2020 [1]. Oncogene activation and/or tumor suppressor gene deactivation results in uncontrolled cell cycle progression and obstruction of apoptotic mechanisms [2]. As opposed to benign tumors, cancerous tumors develop metastasis, which is partially caused by the down-regulation of cell adhesion receptors required for tissue-specific cell–cell attachment and the up-regulation of receptors that facilitate cell mobility. Despite significant scientific and technical advances in the diagnosis and treatment of numerous cancer types over the last decades, we still lack of effective, early stage diagnostic tools and targeted therapeutic approaches.

The combination of tumor abscission and chemotherapy along with or without radiation is widely used for cancer treatment [3,4]. Despite advancements in the field, there are still serious challenges in the treatment of cancer, such as toxicity and side effects that may occur, due to high evasiveness [5]. The limited bioavailability of certain traditional drugs as well as acquired resistance to treatment are additional key limitations of precision therapy. A variety of novel treatments, such as gene therapy, has been arising throughout years; nevertheless, such approaches are currently neglected from widespread use due to their complexity and expensiveness [6]. For example, although vaccines for certain cancer types have been developed, only one has been granted Food and Drug Administration (FDA) approval to date [7]. Moreover, several drug delivery systems based on nanoscale vehicles [8] or autologous cell tissues (i.e., erythrocytes) [9] have been developed, but their efficacy is still under investigation. Therefore, it is crucial to find innovative and applicable therapeutic strategies, auxiliary to current gold standards, for cancer treatment.

Traditionally, one major concern in cancer therapy has been its specificity for cancer tissues without harming surrounding healthy tissues. A very promising approach exhibiting the abovementioned targeted action is hyperthermia treatment (HTT). Indeed, several studies have demonstrated the potential benefits of HTT, including the increased tumor blood flow and oxygenation as well as improved treatment outcomes [10,11]. In recent years, significant advancements have been made in the field of nanotechnology, paving the way for the development and application of nanoparticles as effective hyperthermia agents [12]. Nanoparticles offer unique properties, such as enhanced stability, tunable surface chemistry, and high surface area-to-volume ratio, making them ideal candidates for delivering localized heat to cancerous tissues. Nanotechnology offers tumor-selective drug delivery, thus minimizing toxicity, and has emerged as a very promising field in precision oncology. Novel nanotechnology features could provide meaningful advantages in cancer treatment, such as reinforcement of radiation effect and enhanced bioavailability of therapeutic agents. This paper aims to provide a comprehensive overview of the current landscape in the research of nanoparticle-based cancer hyperthermia, highlighting its potential applications, mechanism of action, and challenges. By examining the latest findings and discussing key studies, we aim to present a comprehensive understanding of the remarkable potential of nanoparticles in revolutionizing cancer therapy.

## 2. Methods and Results

A literature search was conducted in PubMed database as of 16 November 2023, using the following terms: “Cancer AND hyperthermia AND nanoparticles AND cytotoxicity mechanisms”. Of the 50 publications, 44 were of original content, 5 were review articles, and 1 was an editorial. All articles were evaluated for relevance and included in the current work.

Of the 44 original articles, the majority included both in vitro and in vivo experiments. A total of 10 in vivo studies utilized inorganic nanomaterials, such as ferrous or gold; 9 studies included organic nanoparticles, such as liposomes or polymeric nanoparticles; and 2 used carbon-based nanoparticles. A total of 36 in vitro studies included inorganic nanomaterials, 5 included organic nanoparticles, and 1 included carbon-based nanoparticles. The cargo drugs under investigation were doxorubicin, methotrexate, combretastatin, camptosar, nifedipine, and gemcitabine.

## 3. Hyperthermia: Properties and Mechanism of Action

Hyperthermia is defined as treatment with increasing temperatures between 39 °C to 45 °C in order to induce cell death through apoptosis or necrosis [13]. HTT aims to increase intracellular oxidative stress levels, taking advantage of their susceptibility to heating. In terms of surface coverage, three different types of HTT have been established. In whole-body HTT, the temperature increases evenly [14]. The other types are regional and local HTT [15], with the latter being more targeted with respect to the number of affected tissues. Selection is evaluated case-by-case, taking into consideration the location, type, and stage of cancer [16].

Localized HTT is employed for the treatment of cancer that is confined to a particular area. The application of this treatment can be achieved through external, intraluminal, and interstitial methods, which are used to target (i) skin tumors, (ii) subcutaneous tumors, (iii) tumors present within or near the body cavities, and (iv) brain tumors. Heat can be generated using various types of energy sources, such as radiofrequency (RF), ultrasound, and microwaves. Similarly, regional hyperthermia refers to a treatment technique used to heat extensive areas of tissue, including organs, limbs, or body cavities, depending on the type of cancer being treated [10].

Whole-body hyperthermia is used to treat cancers that have spread throughout the body. One critical aspect of this procedure is the introduction of energy into the body while minimizing energy loss. Infrared radiation, radiofrequency energy, and microwave electromagnetic energy are some of the most recent methods used to induce heat that are employed either alone or in combination with each other [10].

One of the primary limitations of both radiation and chemotherapy is the lack of ability to induce cytotoxic responses in tumor cells due to a series of resistance mechanisms [17]. HTT may assist in overcoming these obstacles by inflicting direct double-strand DNA breaks or intercepting DNA repair mechanisms [18,19], thus sensitizing tumor cells to radiation and chemotherapeutic drugs. The application of HTT in tissues generates a series of physiological cell changes, creating a favorable environment for the application of traditional cancer treatments. More specifically, tumor cells become more permeable to drugs [20] due to alterations in membrane properties, such as fluidity [21]. Furthermore, the intracellular levels of ions, such as Ca^2+^, Na^+^, K^+^ and Mg^2+^, are modified [22], resulting in the promotion of cell death signaling. Additionally, increased permeability amplifies the accumulation of fluids and proteins in the tumor microenvironment, leading to a sharp increase in the interstitial fluid pressure (IFP). The later, in turn, exerts pressure on the vessels and reduces vascular perfusion in the tumors [23]. Hyperthermia can exert structural changes in the extracellular matrix by reducing IFP and enhances hydraulic conductivity and lymphatic drainage. This can increase blood perfusion, thus facilitating the intratumoral concentration of NPs [24]. It has been also suggested that modifications to cytoskeletal components may enhance intracellular drug delivery [25] and promote programmed cell death signaling through the induction of (i) alterations in the cytoskeleton-integrin network [26] or (ii) reactive oxygen species (ROS) production in mitochondria [27]. Moreover, nanoparticle-mediated HTT has been reported to increase the apoptosis and necrosis of tumor cells due to collagen fiber damage [28]. Using microCT imaging, one recent study offers insight into NPs migration and intra-tumoral distribution during HT treatment. Increased temperatures promote thermal damage and further induce cell necrosis and apoptosis that contributes to the expansion of interstitial space. This results in augmented tumor porosity that facilitates NP redistribution [29]. Alterations in apoptosis-associated transcription factors, such as heat shock transcription factor 1 (HSF1) [30,31], gene expression modifications [32,33,34], and regulation of anti/pro-apoptotic proteins, such as the anti-apoptotic Bcl-2 protein and the p53 tumor suppressor protein [35,36], have been identified as physiological changes in cancer cells. In fact, utilization of HTT is not utopic as heat shock protein expression may arise [37], thus acting protectively for the tumor by maintaining the growth and survival of cancer cells. However, efforts to optimize the beneficial aspects would be worthwhile.

## 4. Nanoparticles: Definition & Remarks

Nanoparticles (NPs) are defined as solid particles with a diameter range of 10–100 nanometers. They display unique properties, such as a large surface area to volume ratio and the ability to exhibit quantum confinement, making them suitable candidates for a wide range of applications in various fields of biomedicine, such as nanobiotechnology, drug delivery, biosensors, and tissue engineering [38]. The small size of nanoparticles enables them to have greater mobility within the human body when compared to larger materials. Despite stability issues (due to their high surface energy), their unique structural, chemical, mechanical, magnetic, electrical, and biological properties showcase the promising potential of nanomedicine in cancer research [39].

The properties of nanoparticles mainly depend on their size, shape, and surface characteristics. For instance, it has been observed that they exhibit higher reactivity, stronger magnetization, and improved optical and electrical properties compared to their bulk counterparts [40]. Several materials have been used as raw materials for the synthesis of nanoparticles. Some of the most commonly used include metals, such as gold, silver, and platinum; metal oxides, such as titanium dioxide, iron oxide, and zinc oxide; and semiconductors, such as silicon, cadmium selenide, and zinc sulfide [41,42]. Other materials that have been used to synthesize nanoparticles include carbon-based materials, such as graphene and carbon nanotubes [43], as well as biological materials, such as peptides [44], DNA [45], liposomes [46], and micelles [47]. Notably, Doxil^®^, Ambisome^®^, and DepoDur™ are now FDA-approved [48] as they act as direct drug carriers to transport inorganic nanoparticles, including gold or magnetic nanoparticles [49].

NPs have shown diagnostic and prognostic potential in cancer, indicating tumor location and stage of the disease; are considered to be ideal radiosensitizers for radiotherapy due to their high X-ray absorption and unique physicochemical properties [50]; and provide information regarding the efficacy of treatment [51]. These nanoparticles can also carry anticancer therapeutic agents, which can be delivered in precise concentrations via molecular and/or external stimuli. More specifically, tissue-specific accumulation and activation of nanoparticles can be achieved using various methods, such as the application of magnetic fields, light waves, and ultrasounds and the modulation of internal factors, such as pH, temperature, redox potential, and enzymes [52]. This results in their activation only in pathogenic tissues, allowing for the desired therapeutic effect to manifest in a targeted and specific manner [53]. Indeed, nanostructures have been utilized as delivery vehicles, facilitating drug encapsulation or attachment of therapeutic drugs, in order to allow targeted and controlled drug release to specific tissues [54].

**Figure 1:**
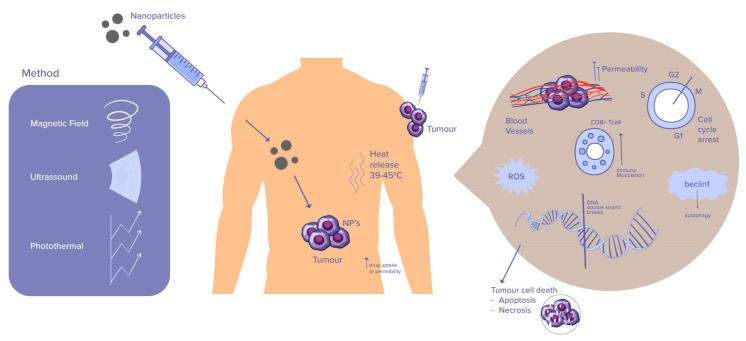
Nanoparticle-mediated, cancer-related hyperthermia mechanisms.

Consequently, NPs have revolutionized the field of medicine due to their unique properties and potential for targeted drug delivery. In addition, recent research has shown that combining nanoparticles with hyperthermia can provide even greater benefits for cancer treatment (as shown in Figure 1). Nanoparticles have garnered considerable attention as versatile platforms for hyperthermia-mediated cancer treatment, as they offer the ability to selectively heat cancerous cells. This application of nanoparticles in hyperthermia-mediated cancer treatment holds tremendous potential to revolutionize oncology and offers new avenues for personalized and targeted therapies.

A variety of external stimuli have been used to initiate the heating process of solid tumors without affecting the surrounding tissues, thus establishing the four main pillars of NP-mediated HTT, including magnetically induced hyperthermia (MIH), photothermal-induced hyperthermia (PIH), radiofrequency-induced hyperthermia (RIH), and ultrasound-induced hyperthermia (UIH), with all of them exhibiting exceptional potentials for cancer treatment.

## 5. Nanoparticle-Associated HTT in Cancer: Current Evidence

As mentioned above, one significant limitation of using HTT as a standalone treatment modality is the indiscriminate warming of surrounding tissues as well as the failure to achieve sufficiently elevated temperatures within the targeted tissue. Ongoing research has focused on the combination of HTT and NPs in an attempt to improve localized cancer treatment. Soon after the first implementation of NP HTT in cancer therapy took place [55], the encapsulation of chemotherapeutic drugs into liposomes and their temperature dependent release occurred [56]. In the following sections, we summarize existing evidence regarding the combination of nanoparticles and hyperthermia. The data obtained from the literature research were categorized based on in vivo (Table 1) and in vitro (Table 2) experiments.

### 5.1. Adenocarcinoma

Only one study on adenocarcinoma was retrieved from our literature review. According to the results, when magnetic Fe_3_O_4_ nanoparticles (MNPs) were subjected to pulsed ultrasound, the cytotoxicity of tumor cells (Ehrlich ascites carcinoma cells [EACs]) was significantly enhanced (both in vitro and in vivo). The treated tumor tissue had areas of apoptotic cells with fragmented chromatin, degraded nuclei and nuclear membranes, and ruptured organelles, suggesting that fundamental changes in cytoskeleton and epigenetics play a pivotal role in the observed cell death. Furthermore, the study suggested that cell viability was time- and MNP-concentration dependent [57].

### 5.2. Bone

The Fe_3_O_4_-doped mesoporous bioactive glass NPs exhibited a hyperthermia effect when exposed to an alternating magnetic field (AMF), indicating potential applications in HTT therapy. Additionally, these NPs demonstrated favorable biocompatibility and low cytotoxicity, making them a safe and promising biomaterial for use in drug delivery for bone tissue regeneration. To further elaborate, in vitro analysis of cell viability using MTT assays of normal human fibroblast (NHFB) cells and an osteosarcoma cell line (MG-63) showed that noticeable anti-proliferative and dose-dependent effects were observed using mitomycin C-loaded NPs (Mc-Fe_3_O_4_-MBG NPs), whereas cell viability was maintained in the NHFB cell line treated with Fe_3_O_4_-MBG [58].

### 5.3. Brain

The surface of nanoplates used in the study by Zhao et al. was engineered with polydopamine, which provided effective photothermal conversion ability. This allowed the increase in localized temperature and acceleration of the intratumoral Fenton-like process in the tumor site arising from U-87 MG cells, a cell line that was isolated from malignant gliomas. A high synergistic effect of laser phototherapy and nanoparticles that promoted cell death was observed both in vitro and in vivo. In fact, cell viability was maintained without laser irradiation, thus presenting the favorable bio-compatibility of NPs. Further analyses demonstrate a slight necrotic response after treatment due to the cytolethal distending toxin (CDT) effect, and nuclear pyknosis, tumor necrosis, and typical apoptosis were observed when combining the two methods [59].

Thermosensitive magnetic liposomes that can be triggered by an alternating magnetic field have been shown to improve the efficacy and reduce the side effects of camptosar in nude mice injected with human primary glioblastoma cells (U87) [60]. A distinct study revealed the same conclusion regarding the efficacy of temozolomide (TMZ), a chemotherapeutic used for high-grade gliomas. TMZ was sheathed into metal core nanoparticles and injected to rats with C6 glioma in combination with alternating magnetic field (AMF) exposure, and the method promoted apoptosis of glioma cells. Moreover, a signaling pathway that induced tumor suppression was identified by western blot analysis and involved suppression of bcl-2 protein and increased levels of Bax protein [61].

### 5.4. Breast

The majority of scientific publications identified focused on the utilization of nanoparticles in conjunction with hyperthermia as a potential therapeutic approach for breast cancer [62,63,64,65,66,67,68,69,70,71,72,73,74,75,76,77,78,79,80,81,82]. All types of exogenous stimuli sources were tested using in vitro assays, while radiofrequency-induced hyperthermia, in particular microwave hyperthermia, has only been tested in vivo in the treatment of chest wall breast cancer recurrence [63]. Current treatment options for this entity are being summarized in a recent review by Youssef et al. Hyperthermia in conjunction with other available treatment modalities, such as surgical resection, radiation therapy, chemotherapy, and immunotherapy, appears to have favorable outcomes in terms of local disease control and prognosis [63]. As the world’s most prevalent cancer type, breast cancer presents an opportunity to evaluate drug release efficacy under both in vitro and in vivo conditions. Cancer cell apoptosis [64,65,66,67] or necrosis [66,67], inhibition of tumor angiogenesis [68], and tumor-targeted and beclin-1-induced autophagy [69,70] are associated with tumor growth inhibition. On the other hand, the enhancement of anti-tumor immunity [71] and increased T cell cytotoxicity in tumor sites [72] have been reported.

Previous research has highlighted doxorubicin (DOX), the most commonly employed chemotherapeutic agent, as a promising candidate for nanoparticle-mediated drug delivery systems [73]. Combined treatment of BALB/c mice with low-toxic porous silicon nanowires (PSi NWs) resulted in tumor cell death following the application of high-intensity ultrasound hyperthermia [66]. Interestingly, various combinations of core materials and coatings, such as gold-liposome [74], citosan@carbon nanotubes-NIPAM [66], mesoporous silica-polydopamine [75], and mesoporous silica@ICG-polyadenine [76], were employed to investigate the in vitro and in vivo efficacy of drug release for DOX-loaded nanoparticles [77,78,79,80,81,82,83,84].

### 5.5. Cervix

In vitro investigations conducted on HeLa and Hep2 cell lines have yielded valuable information regarding the potential applications of hyperthermia induced by photothermal [85] or RF stimulation agents, inducing RF-mediated cell membrane breakdown [86]. Although augmented cell killing rates using different NPs after irradiation were shown, research limitations, such as RF exposure time, intensity, concentration of nanoparticles in cell culture, and incubation time, require further investigation. Furthermore, the release properties of doxorubicin have been evaluated [87,88], demonstrating encouraging outcomes in terms of nanoparticle utilization for the management of cervical cancer. Minimal cytotoxicity was reported when applied individually. On the contrary, interdependent interactions between doxorubicin-coated NPs and AMFs result in low cell viability due to the facilitation of endocytosis in an acidic environment and intracellular release of the drug. Nevertheless, the implied in vivo effects of this treatment approach remain uncertain.

### 5.6. Colon

The utilization of AuNP, as a highly effective sonosensitizer capable of harnessing the thermal and mechanical effects of ultrasound to inflict damage, specifically at the site of the targeted tumor, has been suggested [89]. Tumor metabolic parameters used in PET/CT scans, such as Standardized uptake value (SUV) and total lesion glycolysis (TLG), appeared significantly reduced in treated animals. It has been hypothesized that tumor ablation is caused by the collapse cavitation phenomenon, shock waves and jet formation. Furthermore, the interaction of ultrasound and gold-loaded NPs initiates vascular disruption and tumor necrosis, providing vascular-focused ultrasound therapy.

This thermal and mechanical damage attenuated by NPs was further investigated. Ultrasound-responsive nanomaterials, such as gold nanoparticles (AuNPs), iron oxide nanoparticles (IONPs), and nano-graphene oxide (NGO), have been suggested as viable options to concentrate the energy of acoustic waves on the tumor and trigger localized hyperthermia [90]. It was therefore necessary to consider the combined use of liposome-coated Fe_2_O_3_ nanoparticles encapsulated with combretastatin A4 phosphate (CA4P) [91], a vascular targeting drug that disrupts tumor blood flow [92]. In vivo tumor regression was assessed with MRI using parameters such as Ktrans and extracellular volume (Ve), and the results were corroborated with histological analyses for microvascular density and cellularity. The vascular inhibition effectiveness of the drug was further attenuated with the conjunction of magnetic targeting. Similarly, alginate-coated gold nanoparticles transferring cisplatin demonstrated anti-tumor efficacy in BALB/c mice injected with CT26 colon adenocarcinoma cells by decreasing their metabolic activity, as assessed by PET/CT scan [93].

### 5.7. Liver

The mouse hepatocellular carcinoma cell line (H22) has been used to assess the induction of hyperthermia due to the presence of Ag_2_Se nanodots upon laser irradiation, providing a possible theranostic agent [94]. This was the only in vivo approach found in the literature, whereas in vitro studies shed light to the gold nanoparticle-assisted, RF-induced hyperthermia effect [95] and alternating magnetic field (AMF)-induced hyperthermia in the presence of Fe_3_O_4_ NPs [96] in HepG2 cells. The apoptotic cell ratio was decreased in gold nanoparticle-incorporated and RF-treated cells, implying a protective role of electro-hyperthermia, whereas in the later study, cell viability was significantly reduced upon AMF exposure. Furthermore, drug release examinations provided useful data on the cytotoxic effects of curcumin–nifedipine [97] and DOX [98] when encapsulated in hyperthermia-induced drug delivery systems by promoting apoptosis and the generation of ROS.

### 5.8. Lungs

Recently, in vitro and in vivo evaluation of MnZnFe_3_O_4_-HA NPs showed outstanding activity in lung adenocarcinoma A549 cells. It has been reported that this therapeutic performance is achieved by boosting oxygenation levels and subsequently enhancing the radiofrequency’s effect [99]. The synergistic anticancer effect of MnZnFe_3_O_4_-HA NPs and RIH in vitro and in vivo was well documented based on a compelling increase in apoptosis and necrosis in the cell population, but also by restraining tumor growth and size in histological studies. In the same cell line, the effects of ferum oxide NPs on the release properties of DOX were assessed when AMF [100] or RF [101] was administered. In tumor-xenografted mice, iron-dextran used as a thermosensitizer after RF stimulation completely eradicated cancer caused by the injection of NCI-H460 cells [102]. Radiation-related release of DOX from silica NPs was assessed in in vitro and in vivo experiments, where it suppressed the growth of the carcinoma and prolonged the survival time of the animals [103].

### 5.9. Prostate

Albarqi et al. suggested that the injection of hydrophobic iron oxide nanoparticles intravenously into mice bearing DU145 human prostate carcinoma cell line xenografts efficiently promoted the accumulation of nanoparticles at the tumor site. Exposure to AMF effectively increased the intra-tumor temperature above 42 °C and significantly inhibited prostate cancer growth without exhibiting any toxic effects in normal tissues [104]. Furthermore, Zhu et. al. used microCT scan blood perfusion rates to evaluate the thermal response and transport mechanism of injected NP-mediated hyperthermia in PC3 tumors implanted on the flank of healthy mice [105]. Thermal damage from hyperthermia promotes NP redistribution to the tumor periphery, a phenomenon that minimizes the required treatment time that is originally predicted from traditional protocols. Another recent study investigated the effects of inverse heat transfer, using infrared imaging techniques, on PC3 tumor cells implanted in Balb/c mice [106]. The results from the latter studies may facilitate future experiments by implementing heating variables and intratumoral NP redistribution.

### 5.10. Pancreas

The γ-Fe_2_O_3_ NPs loaded with gemcitabine (GEM) developed by Lafuente-Gómez et al. demonstrated high cytotoxicity in three distinctive pancreatic cancer cell lines (PANC-1, BxPC-3 and MIA Paca-2), displaying different susceptibility to GEM. Thus, the combination of chemotherapeutic agent with MIH definitely contributed to the establishment of a synergistic cytotoxic effect, including the generation of ROS, in all cell lines under evaluation [107].

### 5.11. Sarcoma

One of the latest studies included in this review investigated the possible effects of merging PIH with immunogenic cell death in vivo. This was accomplished by coating MnFe_2_O_4_ NPs with red blood cell membranes, resulting in enhanced survival rates of Swiss Albino mice injected with murine sarcoma cells (S180) [108]. Flow cytometry data clearly demonstrated decreased viability of S180 cells, with the predominance of late apoptosis as a death mechanism in vitro. Furthermore, the antitumor immune response mediated via extraction of HMGB1 and calreticulin suggested ICD under PTT. The same murine sarcoma cancer cell line was used to induce tumors in BALB/c mice in pursuance of highlighting the RF-dependent release of DOX [109]. The theranostic efficacy of C60@Au hybrid nanocomposite loaded with DOX was assessed with in vivo and in vitro studies, showing RF-controlled drug releasing potential, tumor targeting properties, and X-ray imaging abilities.

### 5.12. Skin

Using macromolecules (DNA and cytochrome C) as coating agents of Au-NPs, Park et al. exposed B16 F10 mouse melanoma cells to a PT laser source to induce HTT, which subsequently promoted tumor-selective cell death [110]. These pH-sensitive CytC/ssDNA-AuNPs stimulate high accumulation of AuNPs within the cell. The photothermal therapeutic efficiency of pH-responsive nanoparticles and their ability to induce tumor-selective death were investigated using cytotoxicity tests on normal (MDCK-GFP) and B16F10 cancer cells. B16-F0 tumor mice models were used to assess the UIH effect that was mediated by Yb^3+^/Er^3+^-Zeolite NPs through the production of cytoplasmic ROS and mitochondrial superoxide [111].

**Table 1:** In vivo experiments focusing on the combination of nanoparticles and hyperthermia.

Tumor Model	Method	Core Material	Coating	Cargo	Cell Line	References
**Adenocarcinoma**	UIH ^1^	Fe_3_O_4_	-	-	Swiss albino mice injected with EACs	[57]
**Brain**	PIH ^2^	MnGdO	PDA-PEG	-	Nude mice injected with U-87 MG cells	[59]
**Breast**	MIH ^3^	Fe_3_O_4_	Dextran-folic acid	-	BALB/c mice	[62]
	MIH	Fe_3_O_4_	PAMAM dendrimer	-	Bagg albino strain C (BALB/c) mice	[64,69]
	MIH	MoS_2_/CoFe_2_O_4_	-	-	BALB/c mice tumor	[68]
	PIH	Melanin	-	-	BALB/c nude mice injected with MDA-MB-231 cells	[70]
	PIH	SPNs	PEG	-	BALB/c mice injected with 4T1 cells	[86]
	DR ^4^ PIH	Gold	Liposome	Doxorubicin	BALB/c mice bearing MCF-7 tumors	[78]
	DR PIH	Fluorinated aza-boron-dipyrromethen	-	Doxorubicin	Mice bearing 4T1 tumors	[80]
	DR PIH	Citosan@carbon nanotubes	NIPAM	Doxorubicin	Mice with Luc-4T1 orthotopic tumors	[72]
	DR PIH	Mesoporous silica	Polydopamine	Doxorubicin	BALB/c mice injected with 4T1 cells	[81]
	DR PIH	Mesoporous silica@ICG	Polyadenine	Doxorubicin	Mice bearing 4T1 tumors	[71]
	UIH	nrGO@MSN-ION-PEG	-	-	BALB/c injected with SKBr3	[75]
	DR UIH	Liposome	-	Doxorubicin	BALB/c athymic nude mice injected with MDA-MB-231	[83]
	DR UIH	Liposome	-	Doxorubicin	Mouse 4T1 breast tumor model	[82]
**Colon**	UIH	Gold	-	-	BALB/c mice-bearing CT26 colorectal tumor model	[89]
	UIH	Gold, iron oxide and graphene oxide	-	-	BALB/c mice injected with CT26	[90]
	DR UIH	Fe_2_O_3_	Liposome	Combretastatin A4 phosphate	BALB/c	[91]
**Colorectal**	DR UIH	Gold	Alginate	Cisplatin	BALB/c mice injected with CT26	[93]
**Glioblastoma**	DR MIH	Fe_3_O_4_	Liposome	Camptosar	BALB/c nude mice injected with U-87 cells	[60]
**Glioma**	DR MIH	Fe_3_O_4_	PEG-PBA-PEG	Temozolomide	C6 glioma in rats	[61]
**Liver**	PIH	UCNPs	CS@Ag_2_Se	-	Kunming mice injected with H22 cells	[94]
**Lung**	MIH	MnZnFe_3_O_4_	HA-PEG-PCL	-	A549 subcutaneous tumor xenografts model	[99]
	RIH ^5^	Iron-dextran	-		BALB/c injected with NCI-H460	[102]
	DR RIH	Silica	NIPAM copolymer	Doxorubicin	CBA line mice with lung carcinoma (3LL) tumors	[103]
**Melanoma**	DR UIH	Yb^3+^/Er^3+^ Zeolite	FA-PEG	Doxorubicin	B16-F0 tumor model mice	[111]
**Prostate**	MIH	MnZnFe_3_O_4_	PEG-PCL	-	Nude mice bearing subcutaneous DU145 xenografts	[104]
**Sarcoma**	DR RIH	C60@Au-HBA	PEG	Doxorubicin	BALB/c S180 tumor models	[109]
**Sarcoma**	PIH	MnFe_2_O_4_	Red Blood Cell Membrane	-	Swiss albino mice injected with S180 cells	[108]

^1^ UIH: Ultrasound-induced hyperthermia. ^2^ PIH: photothermal-induced hyperthermia. ^3^ MIH: magnetically induced hyperthermia. ^4^ DR: drug release. ^5^ RIH: radiofrequency-induced hyperthermia.

**Table 2:** In vitro experiments focusing on the combination of nanoparticles and hyperthermia.

Tumor Model	Method	Core Material	Coating	Cargo	Cell Line	References
**Adenocarcinoma**	UIH	Fe_3_O_4_	-	-	Ehrlich ascites carcinoma cells (EACs)	[57]
**Bone**	MIH	Fe_3_O_4_-Bioactive Glass	-	-	Normal human fibroblast (NHFB) and cancer cells (MG-63)	[58]
**Brain**	PIH	MnGdO	PDA-PEG		U-87 MG cells	[59]
**Breast**	MIH	Fe_3_O_4_	Dextran-Folic acid	-	MC4-L2	[62]
**Breast**	MIH	Fe_3_O_4_	PAMAM dendrimer	-	Human breast cancer cell line (MCF7) and human fibroblast cell line (HDF1)	[64,69]
	DR MIH	Fe_3_O_4_	NIPAM-co-DMAEMA	Methotrexate	MCF-7 breast cancer cell line	[77]
	PIH	Melanin	-	-	NIH3T3 cells (ATCC), Hela cells, and MDA-MB-231 cells (ATCC)	[70]
	PIH	Gold	Gold PEG	-	MCF7 and 4T1 cells	[74]
	PIH	SPNs	PEG	-	4T1 and RAW264.7 cells	[84]
	DR PIH	Gold	Liposome	Doxorubicin	MCF-7 breast cancer cell line	[78]
	DR PIH	Gold	NIPAM	Doxorubicin	Hela and MDA-MB-231 cells	[79]
	DR PIH	Fluorinated aza-boron-dipyrromethen	-	Doxorubicin	4T1 cells	[80]
	DR PIH	Citosan@carbon nanotubes	NIPAM	Doxorubicin	4T1 cells	[72]
	DR PIH	Mesoporous silica	Polydopamine	Doxorubicin	4T1 cells	[80]
	DR PIH	Mesoporous silica@ICG	Polyadenine	Doxorubicin	A549 cells	[71]
	RIH	Au@IONPs	-		MCF-7 breast cancer cells	[65]
	RIH	Gold, Iron oxide, Gold@iron oxide	-		Fibroblast (L-929) and breast cancer (MCF-7) cell lines	[66]
	DR RIH	La0.7Sr0.3MnO_3_	Chitosan	Doxorubicin	MCF-7 and MDA-MB-231	[67]
	UIH	nrGO@MSN-ION-PEG	-	-	SKBr3 cell line	[75]
	DR UIH	Liposome	-	Doxorubicin	4T1 mammary carcinoma cells, MCF-7 human breast adenocarcinoma cells, and human umbilical vein endothelial cells (HUVECs)	[82]
	Manual Temperature Swift	Gold	Liposome	Doxorubicin	MDA-MB-231	[76]
**Cervical**	DR MIH	MnZnFe_3_O_4_	Chitosan-g-NIPAM	Doxorubicin	Human cervical cancer cells (HeLa cells)	[87]
	DR MIH	Gadolinium Ferrite	PAMAM	Doxorubicin	HeLa cells	[88]
	PIH	MoO_3_	Cysteine	-	HeLa cells	[85]
	RIH	Silicon NW	-		Hep2 cells	[86]
**Colon**	DR UIH	Fe_2_O_3_	Liposome	Combretastatin A4 phosphate	EA.hy926 cell line	[91]
**Glioblastoma**	DR MIH	Fe_3_O_4_	Liposome	Camptosar	U-87 human primary glioblastoma cell line	[60]
**Liver**	MIH	Fe_3_O_4_	PCL	-	Human liver cancer cells (HepG2)	[96]
	DR MIH	Fe_3_O_4_	PLGA	Curcumin and nifedipine	HepG2 cancer cells	[97]
	PIH	UCNPs	CS@Ag_2_Se	-	A549 cells	[94]
	DR PIH	Chitosan @ ICG	NIPAM	Doxorubicin	HepG2 cancer cells	[98]
	RIH	Gold	-		HepG2 human hepatocellular carcinoma cell line	[95]
**Lung**	MIH	MnZnFe_3_O_4_	HA-PEG-PCL	-	A549 (human lung adenocarcinoma cell line)	[99]
	DR MIH	Fe_3_O_4_	PEG1500	Doxorubicin	Human lung adenocarcinoma (A549)	[100]
	RIH	Iron-Dextran	-		Human lung cancer NCI-H460 cells	[102]
	DR RIH	Iron oxide	Liposome@gold	Doxorubicin	A549	[101]
	DR RIH	Silica	NIPAM copolymer	Doxorubicin	HeLa and HEP2 cells	[103]
**Melanoma**	DR UIH	Yb^3+^/Er^3+^ Zeolite	FA-PEG	Doxorubicin	B16-F0, 4T1, HBE, and U937 cell lines	[111]
**Ovaries**	Manual Temperature Swift	Gold	Liposome	Doxorubicin	SK-OV-3	[76]
**Prostate**	MIH	MnZnFe_3_O_4_	PEG-PCL	-	DU145 human prostate carcinoma cell line and HEK-293 human embryonic kidney cell line	[104]
**Pancreas**	MIH	γ-Fe_2_O_3_	Dextran	Gemcitabine	PANC-1, BxPC-3, and MIA Paca-2	[105]
**Sarcoma**	DR RIH	C60@Au-HBA	PEG	Doxorubicin	MCF-7 cells	[107]
	PIH	MnFe_2_O_4_	Red Blood Cell Membrane	-	S180	[106]
**Skin**	PIH	Gold	DNA & Cytochrome C	-	B16 F10 mouse melanoma cells	[108]

## 6. Future Applications and Challenges

The use of hyperthermia paired with nanoparticle-mediated medication delivery has an upward trajectory in cancer therapy. Preclinical data have demonstrated that the use of nanoparticles as carriers facilitates targeted delivery by increasing blood vessel permeability and allowing controlled chemotherapy release at the tumor [51]. Heterogenous blood perfusion prevents an even temperature distribution within cancerous tissues. A recent study suggests a modified Pennes bioheat equation that integrates perfusion differentiation to detail local variations within the same tumor that was obtained using imaging techniques [112]. This model facilitates treatment planning by clarifying the thermal properties of tissues.

Furthermore, a differentiation in the tumor accumulation of NPs has been described since macrophages and fibroblasts within the tumor microenvironment can affect the nanoparticle distribution [113]. Cell necrosis-induced diffusivity changes and porosity enhancement are two distinct mechanisms that also stimulate nanoparticle migration [29]. It is hypothesized that the NP trajectory is influenced by heat transfer, and thermal damage additionally boosts NP re-distribution within the cancerous tumor [114]. Several heating protocols based on MRI imaging have studied the blood supply of cancerous tumors. These methods have directly linked heating parameters, such as dosage and duration, in an attempt to avoid harming the surrounding normal cells [115]. Recent mathematical developments integrate bioheat and kinetic models in order to combine (i) tissue regeneration under thermal damage and (ii) NP migration and to provide insight into clinical applications of NP-mediated hyperthermia [116].

Moreover, HT further sensitizes cancer cells to chemotherapy, having the advantages of minimal systemic toxicity. Some studies suggest that cancer stem cells are sensitized to radiation therapy by nanoparticle-mediated HT [117], while there is also evidence that hyperthermia can trigger immune-mediated responses in the cancer microenvironment [118].

The integration of hyperthermia into nanoparticle-mediated drug delivery holds great promise as a future strategy for cancer treatment. The optimal dose of magnetic nanoparticles prior to radical prostatectomy or cystoprostatectomy in the treatment of prostate or bladder cancer, respectively, is being currently investigated in the MAGNABLATE I study (NCT02033447). Another ongoing clinical trial investigates the use of hyperthermic intraperitoneal chemotherapy combined with systemic chemotherapy with nab-paclitaxel or cisplatin in pancreatic cancer patients with peritoneal metastasis (NCT04858009).

However, careful observation of the existing data shows substantial variability in tissue and cell line responses to diverse treatment modalities (Table 1 and Table 2). Differences between neoplastic lesions, heterogeneity within the same tissue type, and differences between in vivo studies require further elaboration [119]. Moreover, it is unclear what occurs when NP-mediated HT treatment is administered to humans. While most in vivo studies have evaluated hyperthermia in subcutaneous xenograft models, administering hyperthermia in deep tissues, such as human organs, did not yield consistent findings. On the other hand, the low penetration of the magnetic field narrows the efficacy of the method. However, data from in vitro and animal model studies should be further tested in terms of consistency in humans, as differences in biologic characteristics, as well as the complexity of the human immune and genetic systems, can alter treatment results.

Open questions remain regarding the biocompatibility and/or the possible toxicity that may rise from injecting nanoparticles in healthy tissues. Thus, the evaluation under standard protocols that ensure biosafety must be held in practice [120]. The ISO testing requirements suggest standardized testing methods to evaluate the baseline toxicity of any medical treatment, including assessments of genotoxicity, cytotoxicity, hemocompatibility, tissue tolerance, and pyrogenicity. Indeed, most nanoparticles have been shown to be relatively nontoxic at the required therapeutic doses. For example, gold has been used for many years in the treatment of various medical conditions. Expanding NP-mediated HT research in clinical settings highlights another problem associated with the consistency of large-scale NP production compared with small batches [121]. Moreover, in addition to the appropriate size of nanoparticles being a challenge in manufacturing, high cost is another issue to be mentioned.

In addition, the diversity in NP biodistribution poses challenges that need further clinical interpretation. For example, once in systemic circulation, NPs can be delivered both to the tumor site as well as other human organs. Moreover, the poor vasculature of some cancerous tissues prevents a uniform distribution in tumor core temperature when applying NP-mediated hyperthermia [122]. Thus, both NP delivery and distribution in tumor sites are also two main issues that require further investigation.

Repeatability and standardization of the experimental methods used to investigate the impact of HT should also be further assessed since contradictory results exist in the available literature. Standardized protocols for NP testing, including incubation times, NP concentrations in cells, and radiation parameters, are also pending. Furthermore, dedicated studies assessing pharmacokinetic and pharmacodynamic parameters in addition to safety and efficacy should be designed and carried out.

Finally, one should take into account the ethical and practical considerations that arise from the use of nanotechnology in the treatment of cancer. In addition to nonmaleficence, the potential risks in such work environments should to be further addressed. Moreover, the accumulation and release of nanomaterials could also lead to environmental problems. A further concern before obtaining patient consent relates to the lack of sufficient data to inform participants. Another issue may also be the limited access of some patients to such innovative treatments due to geographical or financial restrictions [123].

## 7. Concluding Remarks

Conventional therapeutic approaches are facing challenges due to tumor aggressiveness and resistance to treatment, leading to a pressing need for novel treatment modalities. Hyperthermia has emerged as a potential candidate, although its use as a single modality is limited. However, it seems that the combination of chemotherapeutic agents delivered using nanoparticle carriers results in synergistic anti-cancer effects and outcomes according to the existing preclinical data. Future studies will highlight the possible clinical applications of this approach and its implementation in everyday clinical practice.

## Data Availability

Not applicable.

## References

[1] Ferlay J., Colombet M., Soerjomataram I., Parkin D.M., Piñeros M., Znaor A., Bray F. (2021). Cancer statistics for the year 2020: An overview. Int. J. Cancer.

[2] Upadhyay A. (2021). Cancer: An unknown territory; rethinking before going ahead. Genes Dis..

[3] Behranvand N., Nasri F., Zolfaghari Emameh R., Khani P., Hosseini A., Garssen J., Falak R. (2022). Chemotherapy: A double-edged sword in cancer treatment. Cancer Immunol. Immunother..

[4] Antoni D., Claude L., Laprie A., Lévy A., Peignaux K., Rivera S., Schick U. (2022). Les essais qui changent les pratiques: Le point en 2022. Cancer/Radiothérapie.

[5] Hauner K., Maisch P., Retz M. (2017). Nebenwirkungen der Chemotherapie. Der Urol..

[6] Couchoud C., Fagnoni P., Aubin F., Westeel V., Maurina T., Thiery-Vuillemin A., Gerard C., Kroemer M., Borg C., Limat S. (2020). Economic evaluations of cancer immunotherapy: A systematic review and quality evaluation. Cancer Immunol. Immunother..

[7] Kantoff P.W., Higano C.S., Shore N.D., Berger E.R., Small E.J., Penson D.F., Redfern C.H., Ferrari A.C., Dreicer R., Sims R.B. (2010). Sipuleucel-T immunotherapy for castration-resistant prostate cancer. N. Engl. J. Med..

[8] Nagaraju G.P., Srivani G., Dariya B., Chalikonda G., Farran B., Behera S.K., Alam A., Kamal M.A. (2021). Nanoparticles guided drug delivery and imaging in gastric cancer. Semin. Cancer Biol..

[9] Castro F., Martins C., Silveira M.J., Moura R.P., Pereira C.L., Sarmento B. (2021). Advances on erythrocyte-mimicking nanovehicles to overcome barriers in biological microenvironments. Adv. Drug Deliv. Rev..

[10] Song C.W., Park H.J., Lee C.K., Griffin R. (2005). Implications of increased tumor blood flow and oxygenation caused by mild temperature hyperthermia in tumor treatment. Int. J. Hyperth..

[11] van der Zee J. (2002). Heating the patient: A promising approach?. Ann. Oncol..

[12] Beik J., Abed Z., Ghoreishi F.S., Hosseini-Nami S., Mehrzadi S., Shakeri-Zadeh A., Kamrava S.K. (2016). Nanotechnology in hyperthermia cancer therapy: From fundamental principles to advanced applications. J. Control. Release.

[13] Szelényi Z., Komoly S. (2019). Thermoregulation: From basic neuroscience to clinical neurology, part 2. Temperature.

[14] Vertree R.A., Leeth A., Girouard M., Roach J.D., Zwischenberger J.B. (2002). Whole-body hyperthermia: A review of theory, design and application. Perfusion.

[15] Chia B.S.H., Ho S.Z., Tan H.Q., Chua M.L.K., Tuan J.K.L. (2023). A Review of the Current Clinical Evidence for Loco-Regional Moderate Hyperthermia in the Adjunct Management of Cancers. Cancers.

[16] Datta N.R., Jain B.M., Mathi Z., Datta S., Johari S., Singh A.R., Kalbande P., Kale P., Shivkumar V., Bodis S. (2022). Hyperthermia: A Potential Game-Changer in the Management of Cancers in Low-Middle-Income Group Countries. Cancers.

[17] Luqmani Y.A. (2005). Mechanisms of drug resistance in cancer chemotherapy. Med. Princ. Pract..

[18] van Oorschot B., Granata G., Di Franco S., Ten Cate R., Rodermond H.M., Todaro M., Medema J.P., Franken N.A. (2016). Targeting DNA double strand break repair with hyperthermia and DNA-PKcs inhibition to enhance the effect of radiation treatment. Oncotarget.

[19] Oei A.L., Vriend L.E., Crezee J., Franken N.A., Krawczyk P.M. (2015). Effects of hyperthermia on DNA repair pathways: One treatment to inhibit them all. Radiat. Oncol..

[20] Gago L., Quiñonero F., Perazzoli G., Melguizo C., Prados J., Ortiz R., Cabeza L. (2023). Nanomedicine and Hyperthermia for the Treatment of Gastrointestinal Cancer: A Systematic Review. Pharmaceutics.

[21] Cividalli A., Cruciani G., Livdi E., Pasqualetti P., Tirindelli Danesi D. (1999). Hyperthermia enhances the response of paclitaxel and radiation in a mouse adenocarcinoma. Int. J. Radiat. Oncol. Biol. Phys..

[22] Yi P.N., Chang C.S., Tallen M., Bayer W., Ball S. (1983). Hyperthermia-Induced Intracellular Ionic Level Changes in Tumor Cells. Radiat. Res..

[23] Leunig M., Goetz A.E., Dellian M., Zetterer G., Gamarra F., Jain R.K., Messmer K. (1992). Interstitial fluid pressure in solid tumors following hyperthermia: Possible correlation with therapeutic response. Cancer Res..

[24] Singh M., Ma R., Zhu L. (2021). Theoretical evaluation of enhanced gold nanoparticle delivery to PC3 tumors due to increased hydraulic conductivity or recovered lymphatic function after mild whole body hyperthermia. Med. Biol. Eng. Comput..

[25] Huang S.H., Yang K.J., Wu J.C., Chang K.J., Wang S.M. (1999). Effects of hyperthermia on the cytoskeleton and focal adhesion proteins in a human thyroid carcinoma cell line. J. Cell. Biochem..

[26] Yonezawa M., Otsuka T., Matsui N., Tsuji H., Kato K.H., Moriyama A., Kato T. (1996). Hyperthermia induces apoptosis in malignant fibrous histiocytoma cells in vitro. Int. J. Cancer.

[27] Terasaki A., Kurokawa H., Ito H., Komatsu Y., Matano D., Terasaki M., Bando H., Hara H., Matsui H. (2020). Elevated Production of Mitochondrial Reactive Oxygen Species via Hyperthermia Enhanced Cytotoxic Effect of Doxorubicin in Human Breast Cancer Cell Lines MDA-MB-453 and MCF-7. Int. J. Mol. Sci..

[28] Piehler S., Wucherpfennig L., Tansi F.L., Berndt A., Quaas R., Teichgraeber U., Hilger I. (2020). Hyperthermia affects collagen fiber architecture and induces apoptosis in pancreatic and fibroblast tumor hetero-spheroids in vitro. Nanomedicine.

[29] Singh M. (2023). Biological heat and mass transport mechanisms behind nanoparticles migration revealed under microCT image guidance. Int. J. Therm. Sci..

[30] Stein U., Jürchott K., Walther W., Bergmann S., Schlag P.M., Royer H.D. (2001). Hyperthermia-induced nuclear translocation of transcription factor YB-1 leads to enhanced expression of multidrug resistance-related ABC transporters. J. Biol. Chem..

[31] Tabuchi Y., Kondo T. (2013). Targeting heat shock transcription factor 1 for novel hyperthermia therapy (review). Int. J. Mol. Med..

[32] Furusawa Y., Tabuchi Y., Takasaki I., Wada S., Ohtsuka K., Kondo T. (2009). Gene networks involved in apoptosis induced by hyperthermia in human lymphoma U937 cells. Cell Biol. Int..

[33] Borkamo E.D., Dahl O., Bruland O., Fluge Ø. (2008). Global gene expression analyses reveal changes in biological processes after hyperthermia in a rat glioma model. Int. J. Hyperth..

[34] Liang H., Zhan H.J., Wang B.G., Pan Y., Hao X.S. (2007). Change in expression of apoptosis genes after hyperthermia, chemotherapy and radiotherapy in human colon cancer transplanted into nude mice. World J. Gastroenterol..

[35] Jiang W., Bian L., Wang N., He Y. (2013). Proteomic analysis of protein expression profiles during hyperthermia-induced apoptosis in Tca8113 cells. Oncol. Lett..

[36] Ahmed K., Zaidi S.F., Mati Ur R., Rehman R., Kondo T. (2020). Hyperthermia and protein homeostasis: Cytoprotection and cell death. J. Therm. Biol..

[37] Shi H., Cao T., Connolly J.E., Monnet L., Bennett L., Chapel S., Bagnis C., Mannoni P., Davoust J., Palucka A.K. (2006). Hyperthermia enhances CTL cross-priming. J. Immunol..

[38] Qamar Z., Qizilbash F.F., Iqubal M.K., Ali A., Narang J.K., Ali J., Baboota S. (2019). Nano-Based Drug Delivery System: Recent Strategies for the Treatment of Ocular Disease and Future Perspective. Recent Pat. Drug Deliv. Formul..

[39] Sultana S., Alzahrani N., Alzahrani R., Alshamrani W., Aloufi W., Ali A., Najib S., Siddiqui N.A. (2020). Stability issues and approaches to stabilised nanoparticles based drug delivery system. J. Drug Target..

[40] Kudr J., Haddad Y., Richtera L., Heger Z., Cernak M., Adam V., Zitka O. (2017). Magnetic Nanoparticles: From Design and Synthesis to Real World Applications. Nanomaterials.

[41] Negrescu A.M., Killian M.S., Raghu S.N.V., Schmuki P., Mazare A., Cimpean A. (2022). Metal Oxide Nanoparticles: Review of Synthesis, Characterization and Biological Effects. J. Funct. Biomater..

[42] Joudeh N., Linke D. (2022). Nanoparticle classification, physicochemical properties, characterization, and applications: A comprehensive review for biologists. J. Nanobiotechnol..

[43] Rhazouani A., Gamrani H., El Achaby M., Aziz K., Gebrati L., Uddin M.S., Aziz F. (2021). Synthesis and Toxicity of Graphene Oxide Nanoparticles: A Literature Review of In Vitro and In Vivo Studies. BioMed Res. Int..

[44] Berillo D., Yeskendir A., Zharkinbekov Z., Raziyeva K., Saparov A. (2021). Peptide-Based Drug Delivery Systems. Medicina.

[45] Hu Q., Li H., Wang L., Gu H., Fan C. (2019). DNA Nanotechnology-Enabled Drug Delivery Systems. Chem. Rev..

[46] Liu P., Chen G., Zhang J. (2022). A Review of Liposomes as a Drug Delivery System: Current Status of Approved Products, Regulatory Environments, and Future Perspectives. Molecules.

[47] Tanbour R., Martins A.M., Pitt W.G., Husseini G.A. (2016). Drug Delivery Systems Based on Polymeric Micelles and Ultrasound: A Review. Curr. Pharm. Des..

[48] Bulbake U., Doppalapudi S., Kommineni N., Khan W. (2017). Liposomal Formulations in Clinical Use: An Updated Review. Pharmaceutics.

[49] Dichello G.A., Fukuda T., Maekawa T., Whitby R.L.D., Mikhalovsky S.V., Alavijeh M., Pannala A.S., Sarker D.K. (2017). Preparation of liposomes containing small gold nanoparticles using electrostatic interactions. Eur. J. Pharm. Sci..

[50] Chen Y., Yang J., Fu S., Wu J. (2020). Gold Nanoparticles as Radiosensitizers in Cancer Radiotherapy. Int. J. Nanomed..

[51] Yao Y., Zhou Y., Liu L., Xu Y., Chen Q., Wang Y., Wu S., Deng Y., Zhang J., Shao A. (2020). Nanoparticle-Based Drug Delivery in Cancer Therapy and Its Role in Overcoming Drug Resistance. Front. Mol. Biosci..

[52] Crucho C.I. (2015). Stimuli-responsive polymeric nanoparticles for nanomedicine. ChemMedChem.

[53] Youns M., Hoheisel J.D., Efferth T. (2011). Therapeutic and diagnostic applications of nanoparticles. Curr. Drug Targets.

[54] Cai X.J., Xu Y.Y. (2011). Nanomaterials in controlled drug release. Cytotechnology.

[55] Jordan A., Wust P., Fähling H., John W., Hinz A., Felix R. (1993). Inductive heating of ferrimagnetic particles and magnetic fluids: Physical evaluation of their potential for hyperthermia. Int. J. Hyperth..

[56] Needham D., Dewhirst M.W. (2001). The development and testing of a new temperature-sensitive drug delivery system for the treatment of solid tumors. Adv. Drug Deliv. Rev..

[57] Shalaby T., Gawish A., Hamad H. (2021). A Promising Platform of Magnetic Nanofluid and Ultrasonic Treatment for Cancer Hyperthermia Therapy: In Vitro and in Vivo Study. Ultrasound Med. Biol..

[58] Ur Rahman M.S., Tahir M.A., Noreen S., Yasir M., Ahmad I., Khan M.B., Ali K.W., Shoaib M., Bahadur A., Iqbal S. (2020). Magnetic mesoporous bioactive glass for synergetic use in bone regeneration, hyperthermia treatment, and controlled drug delivery. RSC Adv..

[59] Zhao Z., Xu K., Fu C., Liu H., Lei M., Bao J., Fu A., Yu Y., Zhang W. (2019). Interfacial engineered gadolinium oxide nanoparticles for magnetic resonance imaging guided microenvironment-mediated synergetic chemodynamic/photothermal therapy. Biomaterials.

[60] Lu Y.-J., Chuang E.-Y., Cheng Y.-H., Anilkumar T.S., Chen H.-A., Chen J.-P. (2019). Thermosensitive magnetic liposomes for alternating magnetic field-inducible drug delivery in dual targeted brain tumor chemotherapy. Chem. Eng. J..

[61] Afzalipour R., Khoei S., Khoee S., Shirvalilou S., Raoufi N.J., Motevalian M., Karimi M.Y. (2021). Thermosensitive magnetic nanoparticles exposed to alternating magnetic field and heat-mediated chemotherapy for an effective dual therapy in rat glioma model. Nanomedicine.

[62] Soleymani M., Khalighfard S., Khodayari S., Khodayari H., Kalhori M.R., Hadjighassem M.R., Shaterabadi Z., Alizadeh A.M. (2020). Effects of multiple injections on the efficacy and cytotoxicity of folate-targeted magnetite nanoparticles as theranostic agents for MRI detection and magnetic hyperthermia therapy of tumor cells. Sci. Rep..

[63] Youssef I., Amin N.P. (2023). Hyperthermia for Chest Wall Recurrence.

[64] Salimi M., Sarkar S., Saber R., Delavari H., Alizadeh A.M., Mulder H.T. (2018). Magnetic hyperthermia of breast cancer cells and MRI relaxometry with dendrimer-coated iron-oxide nanoparticles. Cancer Nanotechnol..

[65] Hadi F., Tavakkol S., Laurent S., Pirhajati V., Mahdavi S.R., Neshastehriz A., Shakeri-Zadeh A. (2019). Combinatorial effects of radiofrequency hyperthermia and radiotherapy in the presence of magneto-plasmonic nanoparticles on MCF-7 breast cancer cells. J. Cell. Physiol..

[66] Nasseri B., Yilmaz M., Turk M., Kocum I.C., Piskin E. (2016). Antenna-type radiofrequency generator in nanoparticle-mediated hyperthermia. RSC Adv..

[67] Kulkarni V.M., Bodas D., Dhoble D., Ghormade V., Paknikar K. (2016). Radio-frequency triggered heating and drug release using doxorubicin-loaded LSMO nanoparticles for bimodal treatment of breast cancer. Colloids Surf. B Biointerfaces.

[68] Alavijeh M., Maghsoudpour A., Khayat M., Rad I., Hatamie S. (2020). Distribution of “molybdenum disulfide/cobalt ferrite” nanocomposite in animal model of breast cancer, following injection via differential infusion flow rates. J. Pharm. Investig..

[69] Salimi M., Sarkar S., Hashemi M., Saber R. (2020). Treatment of Breast Cancer-Bearing BALB/c Mice with Magnetic Hyperthermia using Dendrimer Functionalized Iron-Oxide Nanoparticles. Nanomaterials.

[70] Zhou Z., Yan Y., Wang L., Zhang Q., Cheng Y. (2019). Melanin-like nanoparticles decorated with an autophagy-inducing peptide for efficient targeted photothermal therapy. Biomaterials.

[71] Li X., Wang X., Hua M., Yu H., Wei S., Wang A., Zhou J. (2019). Photothermal-Triggered Controlled Drug Release from Mesoporous Silica Nanoparticles Based on Base-Pairing Rules. ACS Biomater. Sci. Eng..

[72] Bi Y., Wang M., Peng L., Ruan L., Zhou M., Hu Y., Chen J., Gao J. (2020). Photo/thermo-responsive and size-switchable nanoparticles for chemo-photothermal therapy against orthotopic breast cancer. Nanoscale Adv..

[73] Shafei A., El-Bakly W., Sobhy A., Wagdy O., Reda A., Aboelenin O., Marzouk A., El Habak K., Mostafa R., Ali M.A. (2017). A review on the efficacy and toxicity of different doxorubicin nanoparticles for targeted therapy in metastatic breast cancer. Biomed. Pharmacother..

[74] Wang R., Deng J., He D., Yang E., Yang W., Shi D., Jiang Y., Qiu Z., Webster T.J., Shen Y. (2019). PEGylated hollow gold nanoparticles for combined X-ray radiation and photothermal therapy in vitro and enhanced CT imaging in vivo. Nanomedicine.

[75] Chen Y.-W., Liu T.-Y., Chang P.-H., Hsu P.-H., Liu H.-L., Lin H.-C., Chen S.-Y. (2016). A theranostic nrGO@MSN-ION nanocarrier developed to enhance the combination effect of sonodynamic therapy and ultrasound hyperthermia for treating tumor. Nanoscale.

[76] García M.C., Naitlho N., Calderón-Montaño J.M., Drago E., Rueda M., Longhi M., Rabasco A.M., López-Lázaro M., Prieto-Dapena F., González-Rodríguez M.L. (2021). Cholesterol Levels Affect the Performance of AuNPs-Decorated Thermo-Sensitive Liposomes as Nanocarriers for Controlled Doxorubicin Delivery. Pharmaceutics.

[77] Najafipour A., Gharieh A., Fassihi A., Sadeghi-Aliabadi H., Mahdavian A.R. (2021). MTX-Loaded Dual Thermoresponsive and pH-Responsive Magnetic Hydrogel Nanocomposite Particles for Combined Controlled Drug Delivery and Hyperthermia Therapy of Cancer. Mol. Pharm..

[78] He H., Liu L., Zhang S., Zheng M., Ma A., Chen Z., Pan H., Zhou H., Liang R., Cai L. (2020). Smart gold nanocages for mild heat-triggered drug release and breaking chemoresistance. J. Control. Release.

[79] Kwon Y., Choi Y., Jang J., Yoon S., Choi J. (2020). NIR Laser-Responsive PNIPAM and Gold Nanorod Composites for the Engineering of Thermally Reactive Drug Delivery Nanomedicine. Pharmaceutics.

[80] Zhang J., Huang H., Xue L., Zhong L., Ge W., Song X., Zhao Y., Wang W., Dong X. (2020). On-demand drug release nanoplatform based on fluorinated aza-BODIPY for imaging-guided chemo-phototherapy. Biomaterials.

[81] Lei W., Sun C., Jiang T., Gao Y., Yang Y., Zhao Q., Wang S. (2019). Polydopamine-coated mesoporous silica nanoparticles for multi-responsive drug delivery and combined chemo-photothermal therapy. Mater. Sci. Eng. C Mater. Biol. Appl..

[82] Deng Z., Xiao Y., Pan M., Li F., Duan W., Meng L., Liu X., Yan F., Zheng H. (2016). Hyperthermia-triggered drug delivery from iRGD-modified temperature-sensitive liposomes enhances the anti-tumor efficacy using high intensity focused ultrasound. J. Control. Release.

[83] Liang X., Gao J., Jiang L., Luo J., Jing L., Li X., Jin Y., Dai Z. (2015). Nanohybrid liposomal cerasomes with good physiological stability and rapid temperature responsiveness for high intensity focused ultrasound triggered local chemotherapy of cancer. ACS Nano.

[84] Lyu Y., Zeng J., Jiang Y., Zhen X., Wang T., Qiu S., Lou X., Gao M., Pu K. (2018). Enhancing Both Biodegradability and Efficacy of Semiconducting Polymer Nanoparticles for Photoacoustic Imaging and Photothermal Therapy. ACS Nano.

[85] Li Y., Miao Z., Shang Z., Cai Y., Cheng J., Xu X. (2019). A Visible- and NIR-Light Responsive Photothermal Therapy Agent by Chirality-Dependent MoO 3− x Nanoparticles. Adv. Funct. Mater..

[86] Gongalsky M., Gvindzhiliia G., Tamarov K., Shalygina O., Pavlikov A., Solovyev V., Kudryavtsev A., Sivakov V., Osminkina L.A. (2019). Radiofrequency Hyperthermia of Cancer Cells Enhanced by Silicic Acid Ions Released During the Biodegradation of Porous Silicon Nanowires. ACS Omega.

[87] Montha W., Maneeprakorn W., Tang I.M., Pon-On W. (2020). Hyperthermia evaluation and drug/protein-controlled release using alternating magnetic field stimuli-responsive Mn-Zn ferrite composite particles. RSC Adv..

[88] Mekonnen T.W., Birhan Y.S., Andrgie A.T., Hanurry E.Y., Darge H.F., Chou H.Y., Lai J.Y., Tsai H.C., Yang J.M., Chang Y.H. (2019). Encapsulation of gadolinium ferrite nanoparticle in generation 4.5 poly(amidoamine) dendrimer for cancer theranostics applications using low frequency alternating magnetic field. Colloids Surf. B Biointerfaces.

[89] Beik J., Shiran M.B., Abed Z., Shiri I., Ghadimi-Daresajini A., Farkhondeh F., Ghaznavi H., Shakeri-Zadeh A. (2018). Gold nanoparticle-induced sonosensitization enhances the antitumor activity of ultrasound in colon tumor-bearing mice. Med. Phys..

[90] Beik J., Abed Z., Ghadimi-Daresajini A., Nourbakhsh M., Shakeri-Zadeh A., Ghasemi M.S., Shiran M.B. (2016). Measurements of nanoparticle-enhanced heating from 1MHz ultrasound in solution and in mice bearing CT26 colon tumors. J. Therm. Biol..

[91] Thébault C.J., Ramniceanu G., Boumati S., Michel A., Seguin J., Larrat B., Mignet N., Ménager C., Doan B.T. (2020). Theranostic MRI liposomes for magnetic targeting and ultrasound triggered release of the antivascular CA4P. J. Control. Release.

[92] West C.M.L., Price P. (2004). Combretastatin A4 phosphate. Anticancer. Drugs.

[93] Irajirad R., Ahmadi A., Najafabad B.K., Abed Z., Sheervalilou R., Khoei S., Shiran M.B., Ghaznavi H., Shakeri-Zadeh A. (2019). Combined thermo-chemotherapy of cancer using 1 MHz ultrasound waves and a cisplatin-loaded sonosensitizing nanoplatform: An in vivo study. Cancer Chemother. Pharmacol..

[94] Kaimin d., Lei P., Dong L., Zhang M., Gao X., Yao S., Feng J., Zhang H. (2019). In situ decorating of ultrasmall Ag2Se on upconversion nanoparticles as novel nanotheranostic agent for multimodal imaging-guided cancer photothermal therapy. Appl. Mater. Today.

[95] Chen C.-C., Chen C.-L., Li J.-J., Chen Y.-Y., Wang C.-Y., Wang Y.-S., Chi K.-H., Wang H.-E. (2019). Presence of Gold Nanoparticles in Cells Associated with the Cell-Killing Effect of Modulated Electro-Hyperthermia. ACS Appl. Bio Mater..

[96] Hedayatnasab Z., Dabbagh A., Abnisa F., Daud W. (2020). Polycaprolactone-Coated Superparamagnetic Iron Oxide Nanoparticles for In Vitro Magnetic Hyperthermia Therapy of Cancer. Eur. Polym. J..

[97] Sudame A., Kandasamy G., Singh D., Tomy C.V., Maity D. (2020). Symbiotic thermo-chemotherapy for enhanced HepG2 cancer treatment via magneto-drugs encapsulated polymeric nanocarriers. Colloids Surf. A Physicochem. Eng. Asp..

[98] Liu X., He Z., Chen Y., Zhou C., Wang C., Liu Y., Feng C., Yang Z., Li P. (2020). Dual drug delivery system of photothermal-sensitive carboxymethyl chitosan nanosphere for photothermal-chemotherapy. Int. J. Biol. Macromol..

[99] Wang Y., Zou L., Qiang Z., Jiang J., Zhu Z., Ren J. (2020). Enhancing Targeted Cancer Treatment by Combining Hyperthermia and Radiotherapy Using Mn-Zn Ferrite Magnetic Nanoparticles. ACS Biomater. Sci. Eng..

[100] Dabbagh A., Hedayatnasab Z., Karimian H., Sarraf M., Yeong C.H., Madaah Hosseini H.R., Abu Kasim N.H., Wong T.W., Rahman N.A. (2019). Polyethylene glycol-coated porous magnetic nanoparticles for targeted delivery of chemotherapeutics under magnetic hyperthermia condition. Int. J. Hyperth..

[101] Pan A., Jakaria M.G., Meenach S.A., Bothun G.D. (2020). Radiofrequency and Near-Infrared Responsive Core-Shell Nanostructures Using Layersome Templates for Cancer Treatment. ACS Appl. Bio Mater..

[102] Chung H.-J., Kim H.-J., Hong S.-T. (2019). Iron-dextran as a thermosensitizer in radiofrequency hyperthermia for cancer treatment. Appl. Biol. Chem..

[103] Tamarov K., Xu W., Osminkina L., Zinovyev S., Soininen P., Kudryavtsev A., Gongalsky M., Gaydarova A., Närvänen A., Timoshenko V. (2016). Temperature responsive porous silicon nanoparticles for cancer therapy—Spatiotemporal triggering through infrared and radiofrequency electromagnetic heating. J. Control. Release.

[104] Albarqi H.A., Demessie A.A., Sabei F.Y., Moses A.S., Hansen M.N., Dhagat P., Taratula O.R., Taratula O. (2020). Systemically Delivered Magnetic Hyperthermia for Prostate Cancer Treatment. Pharmaceutics.

[105] Singh M., Gu Q., Ma R., Zhu L. (2020). Heating Protocol Design Affected by Nanoparticle Redistribution and Thermal Damage Model in Magnetic Nanoparticle Hyperthermia for Cancer Treatment. J. Heat Transf..

[106] Singh M., Flores H., Ma R., Zhu L. Extraction of Baseline Blood Perfusion Rates in Mouse Body and Implanted PC3 Tumor Using Infrared Images and Theoretical Simulation. Proceedings of the Summer Biomechanics, Bioengineering and Biotransport Conference.

[107] Lafuente-Gómez N., Milán-Rois P., García-Soriano D., Luengo Y., Cordani M., Alarcón-Iniesta H., Salas G., Somoza Á. (2021). Smart Modification on Magnetic Nanoparticles Dramatically Enhances Their Therapeutic Properties. Cancers.

[108] Sousa-Junior A.A., Mello-Andrade F., Rocha J.V.R., Hayasaki T.G., de Curcio J.S., Silva L.D.C., de Santana R.C., Martins Lima E., Cardoso C.G., Silveira-Lacerda E.D.P. (2023). Immunogenic Cell Death Photothermally Mediated by Erythrocyte Membrane-Coated Magnetofluorescent Nanocarriers Improves Survival in Sarcoma Model. Pharmaceutics.

[109] Shi J., Chen Z., Wang L., Wang B., Xu L., Hou L., Zhang Z. (2016). A tumor-specific cleavable nanosystem of PEG-modified C60@Au hybrid aggregates for radio frequency-controlled release, hyperthermia, photodynamic therapy and X-ray imaging. Acta Biomater..

[110] Park S., Lee W.J., Park S., Choi D., Kim S., Park N. (2019). Reversibly pH-responsive gold nanoparticles and their applications for photothermal cancer therapy. Sci. Rep..

[111] Zheng L., Zhang Y., Lin H., Kang S., Li Y., Sun D., Chen M., Wang Z., Jiao Z., Wang Y. (2020). Ultrasound and Near-Infrared Light Dual-Triggered Upconversion Zeolite-Based Nanocomposite for Hyperthermia-Enhanced Multimodal Melanoma Therapy via a Precise Apoptotic Mechanism. ACS Appl. Mater. Interfaces.

[112] Singh M. (2024). Modified Pennes bioheat equation with heterogeneous blood perfusion: A newer perspective. Int. J. Heat Mass Transf..

[113] Wu P., Han J., Gong Y., Liu C., Yu H., Xie N. (2022). Nanoparticle-Based Drug Delivery Systems Targeting Tumor Microenvironment for Cancer Immunotherapy Resistance: Current Advances and Applications. Pharmaceutics.

[114] Singh M., Ma R., Zhu L. (2021). Quantitative evaluation of effects of coupled temperature elevation, thermal damage, and enlarged porosity on nanoparticle migration in tumors during magnetic nanoparticle hyperthermia. Int. Commun. Heat Mass Transf..

[115] Singh M., Singh T., Soni S. (2021). Pre-operative Assessment of Ablation Margins for Variable Blood Perfusion Metrics in a Magnetic Resonance Imaging Based Complex Breast Tumour Anatomy: Simulation Paradigms in Thermal Therapies. Comput. Methods Programs Biomed..

[116] Singh M. (2022). Incorporating vascular-stasis based blood perfusion to evaluate the thermal signatures of cell-death using modified Arrhenius equation with regeneration of living tissues during nanoparticle-assisted thermal therapy. Int. Commun. Heat Mass Transf..

[117] Haque M., Shakil M.S., Mahmud K.M. (2023). The Promise of Nanoparticles-Based Radiotherapy in Cancer Treatment. Cancers.

[118] Zhang Y., Li Z., Huang Y., Zou B., Xu Y. (2023). Amplifying cancer treatment: Advances in tumor immunotherapy and nanoparticle-based hyperthermia. Front. Immunol..

[119] Zhang W., Kohane D.S. (2021). Keeping Nanomedicine on Target. Nano Lett..

[120] (2017). Biological Evaluation of Medical Devices—Part 22: Guidance on Nanomaterials.

[121] Yusefi M., Shameli K., Su Yee O., Teow S.Y., Hedayatnasab Z., Jahangirian H., Webster T.J., Kuča K. (2021). Green Synthesis of Fe_3_O_4_ Nanoparticles Stabilized by a Garcinia mangostana Fruit Peel Extract for Hyperthermia and Anticancer Activities. Int J. Nanomed..

[122] Johannsen M., Thiesen B., Wust P., Jordan A. (2010). Magnetic nanoparticle hyperthermia for prostate cancer. Int. J. Hyperth..

[123] Wasti S., Lee I.H., Kim S., Lee J.H., Kim H. (2023). Ethical and legal challenges in nanomedical innovations: A scoping review. Front. Genet..

